# Prevalence, Trends, and Determinants of Double Burden of Malnutrition Among the Mother–Child Dyads of Pakistan

**DOI:** 10.1155/ijpe/8816802

**Published:** 2025-07-09

**Authors:** Asif Khaliq, Muhammad Salman Godil, Shafaq Taseen, Izzan Ahmed Usmani, Tasin Safwath Chowdhury

**Affiliations:** ^1^School of Public Health and Social Work, Queensland University of Technology, Kelvin Grove, Queensland, Australia; ^2^Department of Medicines, Jinnah Sindh Medical University, Karachi, Sindh, Pakistan; ^3^Department of Paediatrics and Child Health, The Aga Khan University, Karachi, Sindh, Pakistan; ^4^Department of Medicines, Dow International Medical College, Karachi, Sindh, Pakistan; ^5^Department of Statistics, Jahangirnagar University, Dhaka, Dhaka Division, Bangladesh

**Keywords:** determinants, double burden, household, malnutrition, Pakistan, prevalence, trends

## Abstract

**Background:** Double burden of malnutrition (DBM) is a complex nutritional phenomenon, where an individual or a household simultaneously face two contradictory forms of nutritional adversities. Studies on assessing the prevalence, trends, and determinants of DBM at household in Pakistan are scarce, so this study measured the prevalence, trends, and determinants of DBM at household level using nationally representative datasets.

**Methods:** Data of last two waves of Demographic and Health Surveys (DHS) of Pakistan conducted in 2012–2013 and 2017–2018 were used to assess the DBM prevalence, trends, and determinants at the household level. Data of mother–child dyads aged between 15 and 49 years and 0 and 59 months was included, while data with incomplete anthropometry or anthropometric outliers was excluded.

**Results:** Across two survey periods, there has been a significant decrease in pediatrics stunting from 45% to 37.3%, while a rapid proliferation of maternal obesity from 45% to 52.3% was reported. Similarly, the prevalence of DBM at household level was 17.3% in 2012-2013, which increased to 18.9% in 2017–2018. Among different provinces of Pakistan, Baluchistan, Khyber Pakhtunkhwa (KPK) and Federally Administered Tribal Areas (FATA) have significantly higher prevalence of DBM at household than Islamabad Capital Territory (ICT). Factors such as child age, birth order, and socioeconomic status were significantly associated with DBM in Pakistani households.

**Conclusion:** DBM in Pakistan is a complex and chronic challenge, which affects almost one-fifth of households. There is need to urgently address the issues of DBM and to improvise nutrition specific and nutrition sensitive interventions to curtail the issues pertaining to DBM at household.

## 1. Introduction

Malnutrition persists as a pressing global public health challenge, contributing significantly to a range of diet-related noncommunicable diseases. Over the past few decades, there has been a noteworthy decline in undernutrition worldwide, accompanied by a simultaneous increase in the prevalence of overweight and obesity [1]. The phenomenon of sudden decline in undernutrition cases alongside a rapid proliferation of overnutrition has been observed globally and is termed as *nutritional transition* [1, 2]. In general, a transition in dietary habits from traditional low-fat and high-fiber diets to modern high-energy and low-fiber diets and notable declines in physical activity contributes to nutritional transition [1, 3, 4]. These changes in dietary habits are not confined to an individual or community; rather, it has been transcended across the globe. Thus, leading to a complex phenomenon commonly referred to as double burden of malnutrition (DBM) [5, 6].

The complexity of DBM is observed at community, household, and individual levels [6–10]. At the household level, DBM manifests as the coexistence of contrasting forms of malnutrition among mother–child dyads [11, 12]. The emergence of DBM at the household level poses a significant challenge, particularly in low- and middle-income countries (LMICs) [12, 13]. Understanding DBM at the household level is crucial because DBM at the household level reflects broader societal and environmental factors that influence dietary patterns and nutritional outcomes. However, research on the impact of nutrition-specific and nutrition-sensitive interventions on DBM remains limited, despite the endorsement of double-duty actions by the World Health Organization (WHO) [6].

Pakistan is a LMIC, where malnutrition is still considered as an endemic issue, specifically among children below 5 years and women of reproductive age (WRA) [1]. Since 1947, Pakistan has participated in four National Nutritional Surveys (NNS), four Demographic and Health Surveys (DHS), and four Multiple Indicator Cluster Surveys (MICS) for assessing the health and nutritional profile of young children and WRA. All the NNS, DHS, and MICS implemented in Pakistan have examined the nutrition profile at the community level. However, the nutrition profile at the household level has not yet investigated [14]. Exploring the nutrition profile of mother–child's dyads at the household level will provide an integrated approach to the researcher, policy makers, and healthcare providers to address the specific nutritional needs of mothers and children, thereby improving their health outcomes. Thus, this study was designed to examine the prevalence, trends, and determinants of DBM among mother–child dyads in Pakistan using the datasets of last two waves of DHS conducted in 2012–2013 and 2017–2018.

## 2. Methodology

### 2.1. Datasets

In this study, secondary data analysis of DHS conducted in Pakistan was carried out. Out of four DHS surveys, data of the last two waves conducted in 2012–2013 and 2017–2018 was used for examining the prevalence, trends, and determinants of DBM at household level. The data in each DHS were collected by means of different survey questionnaires and were retrieved in the data repository of the DHS program. For assessing the DHS dataset, a formal registration of the project and its objectives was submitted to the DHS program data repository.

### 2.2. Study Population and Eligibility Criteria

In each DHS, WRAs aged between 15 and 49 years were approached via multistage stratified cluster sampling method. Children under 5 years of age were subsampled. This study included all the mother–child dyads aged between 15–49 years and 0–59 months, respectively [12]. Data of nonpregnant mothers, who have at least one child aged between 0 and 59 months was included. However, data of mother–child dyads either with incomplete and/or invalid anthropometric record was excluded.

### 2.3. Measurement of Outcome Variables

The outcome variable of this study was DBM at the household level among mother–child dyads. To assess DBM at the household level, we considered maternal overweight/obesity and pediatric stunting.

In this study, maternal overweight/obesity was measured through body mass index (BMI). For assessing the maternal BMI status, we considered Asian BMI cut-offs range rather than the standard BMI cut-off ranges, because Pakistan is an Asian country, and the Asian population have smaller muscle mass compared to the western population. Moreover, Asians at normal BMI have twice folds higher risk of developing diabetes and other metabolic disorders [15]. Hence, in this study, we considered BMI ranged between 18.5 and 22.9 kg/m^2^ as a normal healthy BMI [15, 16]. However, mother's having BMI exceeding 22.9 kg/m^2^ were classified as overweight/obese rather than those exceeding 25.0 kg/m^2^, while the rest were classified either as underweight (BMI < 18.5 kg/m^2^) [12, 15, 16].

Pediatric stunting manifest impaired child growth and was assessed by measuring a child's height or length in relation to their age and comparing it to standardized growth charts. Children with height-for-age (HAZ) score value of ≤ −2.00 S. D were identified as stunted, while rest were categorized as healthy children having HAZ score > −2.00 S. D [11–14].

Based on the nutrition profile of mother and child, this study has identified four different types of households: (a) *healthy households,* where both child and mother were healthy, (b) *partially healthy households*, in which the household has either a healthy child with an underweight or overweight/obese mother or vice versa, (c) *undernourished households,* where the mother was underweight and the child was stunted, and (d) *households with DBM,* where the mother was overweight/obese and the child was stunted.

From these four household types, two major categories of households were created:

1. Households with DBM.
2. Households without DBM.

### 2.4. Study Covariates

The proposed covariates considered for the analysis were adopted after reviewing different literature [14, 17] and include *child factors*—biological age (in months), sex (male or female), birth order number (index child and subsequent child), and birth size (small, average, large). However, we excluded birth weight from the analysis because over one-third of births in Pakistan occur at home, where infant weight is typically not measured. Among the children with recorded birth weights, more than half of the mothers did not recall their child's birth weight. Among the *maternal factors*, maternal age (below 20 years, 20–34 years, 35 years, or more), maternal education (none, primary, secondary, and higher), maternal occupation (working and not working), maternal BMI (underweight, normal weight, and overweight/obese), last birth cesarean section (yes and no), and marital status (married or divorced/widowed/separated) were included. Factors such as socioeconomic status (poorest, poorer, middle, richer, and richest), family size (small sized family, medium sized family, and large-sized family), place of delivery (home and healthcare center), region (urban and rural), and province (Punjab, Sindh, Baluchistan, Khyber Pakhtunkhwa (KPK), Gilgit Baltistan (GB), Azad Jammu Kashmir (AJK), Federally Administered Tribal Areas (FATA), and Islamabad Capital Territory (ICT)) were included among the household and community factors. The family size was the continuous variable, which was converted into the categorical variable by creating three categories: small sized family, medium sized family, and large-sized family. The average family size in Pakistan is 9.51 ± 4.94. Considering the average family size of Pakistani population, we considered small sized family with family member of 0–4 family members, medium sized family having family members between 5 and 9, and large-sized family with family members consisting of 10 or more members.

### 2.5. Statistical Analysis and Data Management

**Figure 1:**
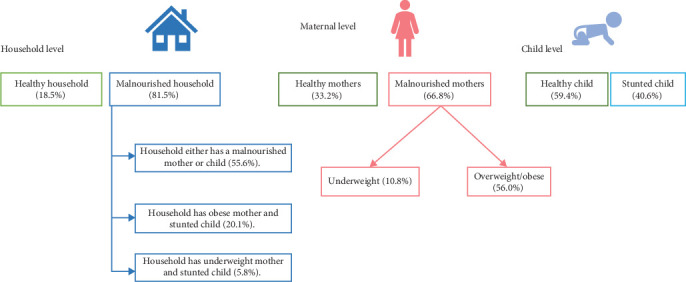
Nutrition profile of the study sample.

**Figure 2:**
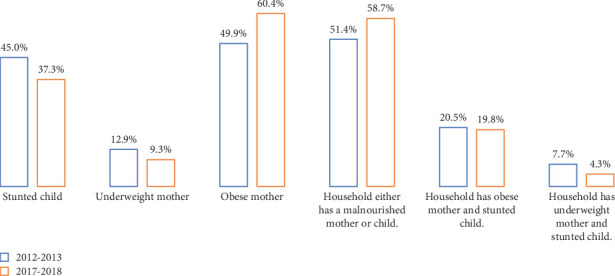
Nutrition profile of the study sample across two survey periods.

The data of this study was obtained from the data repository of DHS. The variables from each dataset were screened thoroughly, and based on the objectives of this study, a list of variables was selected. After variable selection, a preliminary analysis of each dataset was performed. Following preliminary analysis, the data from each dataset was analyzed descriptively. In the descriptive analysis, characteristics of various covariates from each dataset were assessed by calculating the percentages and mean ± standard deviation (Table 1). Furthermore, prevalence and trends of various forms of nutritional disorders among mother–child dyads were also assessed descriptively (Figures 1 and 2). However, the determinants of DBM among the mother–child dyads of Pakistan were analyzed inferentially. For performing inferential analysis, unordered bivariate logistic regression was performed because the outcome variable has two categories: *households with DBM* and *households without DBM*. The relationship of the outcome variable was first measured for each individual variable (Supporting Information (available here)); variables with *p* value ≤ 0.05 showed a significant relationship with the outcome variable. Later, the relationship of the study outcome was analyzed by following the principles of the backward elimination method, in which we considered those variables with *p* value ≤ 0.05 (Table 2). The data was analyzed using the Jamovi software.

## 3. Result

### 3.1. Demographic Characteristics of the Study Sample

In this study, the demographic characteristics of the study sample, drawn from the PDHS conducted in 2012–2013 and 2017–2018, were analyzed. The combined dataset comprised 6198 children, with 2609 from the 2012-2013 survey and 3589 from the 2017-2018 survey. Across two periods, the average age of children was approximately 29 months, with no statistically significant difference observed between the two time points (*p* > 0.05). Gender distribution also remained consistent, with a slightly higher proportion of male children (48.7%) compared to female children (51.3%) (*p* > 0.05).

Regarding maternal factors, the mean age of mothers was 29.5 ± 6.18 years, of which over 75% were of age range 25–34 years. Compared to 2012–2013 survey, the 2017–2018 survey reported a significant improvement in the maternal education (*p* < 0.05), while a notable decline in maternal employment was observed in 2017–2018 survey (*p* < 0.05). Furthermore, a significant increase in the trend of cesarean section deliveries from 13.9% in 2012–2013 to 20.2% in 2017–2018 (*p* < 0.05) was reported.

In terms of household factors, there were shifts in the distribution across wealth index categories, with a decrease in the proportion of households classified as poorest and richest, and an increase in middle wealth categories (*p* < 0.05). Over half of the participants had a medium-sized family consisting of 5–9 family members; however, less than 10% of the households had small-sized families consisting of 0–4 family members. Around 60% of deliveries occurred in health centers rather than at home, with no significant change observed over time (*p* > 0.05). The distribution of households in urban and rural areas also remained relatively stable across both survey periods (*p* > 0.05).

### 3.2. Nutrition Profile of the Study Sample

The nutrition profile of the study sample, analyzed across two periods (2012–2013 and 2017–2018), reveals significant insights into child and maternal nutrition, as well as household nutritional status. The mean HAZ score of children was −1.61 ± 1.78, indicating a high prevalence of stunting (59.4%). The mean BMI of mothers was 24.44 ± 5.26 kg/m^2^, with 33.2% falling within the normal BMI range, 56% being overweight/obese, and 10.8% being underweight. Based on the household nutritional status, 81.5% of households were classified as malnourished (Figure 1).

Across two survey periods, the mean HAZ improved from −1.75 ± 1.89 in 2012–2013 to −1.49 ± 1.68 in 2017–2018, with a corresponding decrease in the prevalence of stunting from 45% to 37.3%. These findings suggest progress in addressing childhood undernutrition in Pakistan. However, the mean BMI increased from 23.72 ± 4.99 kg/m^2^ in 2012–2013 to 24.91 ± 5.37 kg/m^2^ in 2017–2018. The distribution of maternal nutrition profiles shifted, with the proportion of underweight mothers decreasing from 12.9% to 9.3%. Meanwhile, the percentage of mothers with normal weight decreased from 37.2% to 30.5%, and the proportion of overweight or obese mothers increased significantly from 49.9% to 60.4%. This translates to a significant change in the trends of maternal nutritional profile across two survey periods (*p* < 0.05). Similarly, the distribution of malnourished households reported in this study increased from 79.6% to 82.8%. Further breakdown of household nutritional status shows that households with either a malnourished mother or child increased from 51.4% to 58.7%. The prevalence of households experiencing DBM (households with an obese mother and a stunted child) slightly increased from 17.6% to 18.9%, while those with an underweight mother and a stunted child decreased from 7.7% to 4.3% (Figure 2).

### 3.3. Determinants of DBM at Household Level of Pakistan

The analysis revealed a statistically significant positive association between child age and DBM (OR: 1.01, 95% CI: 1.00–1.02). This indicates that for each unit increase in child age, the odds of experiencing DBM increased by a factor of 1.01, highlighting that older children were at a higher risk of being affected by the DBM. Between male and female children, the female children have significantly lower odds of DBM (OR: 0.86, 95% CI: 0.77–0.97) compared with their male counterparts. This suggests that male children were more susceptible to experiencing stunting than female children do.

The presence of a recent birth in the household was associated with lower odds of DBM (OR: 0.67, 95% CI: 0.59–0.77). A protective effect of maternal education against DBM was observed, with higher levels of education associated with reduced odds of DBM. For instance, mothers with higher education had significantly lower odds of experiencing DBM compared to those with no education (OR: 0.60, 95% CI: 0.47–0.77).

The households with richest wealth category having the lowest odds of DBM (OR: 0.65, 95% CI: 0.51–0.82) compared with the poorest households. Significant regional variations in DBM prevalence were observed across different provinces, with certain provinces exhibiting significantly higher odds of DBM compared to others (Table 2).

## 4. Discussion

This research evaluated the prevalence, trends, and determinants of the DBM at the household level in Pakistan using representative datasets from the PDHSs in 2012–2013 and 2017–2018. In Pakistan, more than 40% of children under the age of five are experiencing stunted growth, making it the highest rate [18] in South Asia. Countries with high stunting rates are adversely affected by DBM [19]. The prevalence and trends of DBM in Pakistan have not been quantified by numerous studies conducted in past [20, 21]. Individual country analysis is crucial for understanding the prevalence and impact of DBM within specific populations, as it allows for tailored interventions and policies to address the unique challenges faced by each country.

The nutrition profile of the study, revealed an improvement in average child HAZ, indicating growth, with a decrease in the proportion of stunted children and an increase in the percentage of children with a normal nutrition profile, resulting in 59.4% of children having a normal nutrition profile and 40.6% being stunted overall. Determining the precise cause of the changing pattern in pediatric nutrition status throughout these surveys presents a formidable challenge. We hypothesize several causes for the trend's turnaround during this period. Maternal characteristics, household economic status, maternal education level, food security, household wealth index, access to clean water, sanitation, and rural versus urban place of residence all influence children's nutritional status [22–25]. The variations that were noted between the two survey periods may be accounted for in part by shifts in the socioeconomic attributes of the participants under investigation. Compared to the previous PDHS of 2012–2013, the 2017–2018 PDHS reported significant improvements in socioeconomic indicators, including maternal education, sanitary sanitation facilities, constructed housing infrastructure, and urban influx. A robust correlation was also observed between socioeconomic development and improved health and nutritional outcomes, particularly in rural areas, where enhanced socioeconomic conditions can lead to better access to nutritious food and healthcare services [26, 27].

The average maternal BMI exhibited an upward trend over the years, indicating progressively higher BMI values. This study reported a significant increase in the proportion of overweight or obese mothers in the 2017–2018 survey, compared with the former survey of 2012–2013. Additionally, there was an increase in the prevalence of households exhibiting DBM. Maternal nutrition is crucial for a child's long-term growth and wellness, affecting organ development, cognitive function, and immune system resilience [28]. Malnutrition in mothers can lead to negative outcomes such as low birth weight, poor neurodevelopment, and increased susceptibility to chronic diseases. Maternal nutrition-focused integrative interventions are crucial for achieving optimum results in infant development. Nutrition education programs can promote optimal child growth, better health, and reduce stunting. Empowering mothers through nutrition education can address poor feeding practices and improve their ability to provide adequate nutrition for children. Improving maternal nutrition through prenatal supplementation, nutrition education, and balanced diets can significantly improve child health outcomes [29, 30].

In Pakistan, the prevalence of households experiencing a DBM increased over the years. Factors contributing to this trend include high food insecurity, energy-dense food consumption, limited access to water and healthcare services, maternal education, older mother's age, higher income households, lower maternal education, and having more than four household members [31–33]. These sociodemographic factors, along with maternal characteristics, contribute to the prevalence of DBM. To end malnutrition, the millennium development goals (MDGs) have shifted focus from hunger to nutrition, with the Sustainable Development Goals (SDGs). Policymakers are now focusing on double-duty actions, which involve interventions, programs, and policies that can reduce the risk of undernutrition, overweight, and obesity by leveraging the coexistence of multiple forms of malnutrition and their shared drivers to offer integrated solutions [34]. National dietary guidelines can guide policies to promote high diet quality and reduce undernutrition, overweight, and obesity. Humanitarian aid and emergency nutrition programs can support nutritious diets.

Child age and sex significantly increased the odds of DBM in Pakistan; a similar likelihood of DBM among mother–child pairs is also found in India and Bangladesh [33, 35]. Older children and male children showed increased odds of experiencing DBM, suggesting that as children grow older, the likelihood of facing DBM rises. To reduce DBM in families with multiple children, promote family planning and provide nutritional supplements and fortified foods, ensuring optimal spacing between births. Following that, higher maternal education significantly protects against DBM in the case of Pakistan. However, higher maternal education has a mixed impact on DBM across different countries. In Nepal, educated mothers have a lower risk of DBM, while a study in Myanmar found that women with secondary education had a higher prevalence of being overweight, potentially contributing to DBM at the household level [36–38]. The protective or risk factor role of maternal education in DBM at the household level varies among countries, emphasizing the complex relationship between education and DBM. Therefore, longitudinal and intervention studies are needed to provide conclusive evidence on the protective role of maternal education against DBM.

Socioeconomic status is a crucial determinant that significantly impacts the DBM. In the present study, wealthier households generally have lower odds of DBM. In the same manner, in Sri Lanka, households with higher wealth or education have lower odds of having underweight children [39], while in other South Asian countries, households with higher wealth or education are more likely to have overweight children [12, 31]. However, the study also found a differential burden of overnutrition in South Asian countries, with households of higher wealth or education being less likely to have children overweight, particularly in Pakistan. Low socioeconomic status is linked to the DBM due to factors like limited access to healthy foods, inadequate care practices, and poor environmental conditions. In Bangladesh, the DBM is significantly influenced by socioeconomic status, with wealthier households having higher prevalences of maternal overweight and child undernutrition [12, 40].

The data indicates significant regional and provincial variations in the odds of experiencing DBM. Households in rural areas consistently have higher odds compared to urban areas; this highlights the persistent disparity between rural and urban areas in terms of nutritional challenges. Provincial differences are also notable. In the initial dataset, Baluchistan stands out with significantly higher odds of DBM compared to another province. In the 2017–2018 dataset, the odds for Baluchistan remain elevated, though not as high as previously; but when considering the combined data, Baluchistan and FATA consistently exhibit higher odds of DBM, indicating persistent regional challenges. This research highlights the intricate and diverse situation of nutritional health in various regions and provinces, emphasizing the need for focused interventions that tackle the distinct variables that contribute to malnutrition in these places.

### 4.1. Strengths and Limitations of the Study

This study is the first to assess the relationship of the DBM at the household level in Pakistan. It fills a significant gap in the existing literature and provides a foundation for future research in this area. The nationally representative datasets used in this study are comprehensive, including a wide range of variables relevant to DBM, allowing for a thorough analysis of the factors contributing to DBM at the household level. Data in this study was collected via a multistage stratified sampling technique, and this method of sampling has reduced the potential for sampling bias by adequately representing different subgroups within the population.

Despite great significance, the study also has limitations: The data was collected at a single point in time, cross-sectional in nature, which limits the ability to infer causal relationships between variables. Future studies are encouraged to conduct detailed longitudinal studies to better understand the temporal dynamics of DBM. The findings of this study may not fully reflect the current situation because this study failed to represent statistics of FATA and AJK at both time points. Thus, the absence of data from these areas should be considered while interpreting the results. As with any study relying on survey data, there is a possibility of reporting bias. Respondents might underreport or overreport certain behaviors or conditions due to social desirability, recall bias, or misunderstanding of questions. Although the study moves the knowledge frontier by examining DBM at the household level, some methodological limitations may exist that prevent full control for confounding factors. Factors such as cultural practices, regional dietary habits, and local healthcare accessibility might also influence DBM and need to be considered in future research. Incorporating qualitative methods in the future could provide deeper insights into the lived experiences and contextual factors influencing household nutrition dynamics. While the study provides valuable insights, it does not directly address how current policies and interventions are impacting DBM. Future research could focus on evaluating the effectiveness of existing programs and identifying gaps that need to be addressed.

## 5. Conclusion

DBM at household level is a pressing issue, which affects both the mother and child simultaneously, but in opposite direction. Managing DBM at household level is not simple and straight forward, because it requires double duty of action. In order to combat DBM at household level, the policy makers and program managers need to devise interventions in such a way that it could treat and prevent the emergence of both concurrent undernutrition and overnutrition in an individual and household.

## Figures and Tables

**Table 1:** Demographic characteristics of the study sample.

**Variables**	**Categories**	**2012–2013 (** **n** = 2609**)**	**2017–2018 (** **n** = 3589**)**	**Total (** **n** = 6198**)**
*Child factor*				
Child age		29.4 ± 17.5	28.6 ± 17.7	29.0 ± 17.7
Child sex	Male	1337 (51.2%)	1841 (51.3%)	3020 (48.7%)
	Female	1272 (48.8%)	1748 (48.7%)	3178 (51.3%)
Birth order	Index child	573 (22%)	864 (24.1%)	1437 (23.2%)
	Subsequent child	2036 (78%)	2725 (75.1%)	4761 (76.8%)
Size of birth	Small	471 (18.1%)	597 (16.7%)	1068 (17.2%)
	Average	1918 (73.7%)	2713 (75.6%)	4648 (75%)
	Large	210 (8.1%)	272 (7.5%)	482 (7.8%)

*Mother factors*				
Mother age		29.61 ± 6.26	29.32 ± 6.12	29.5 ± 6.18
Mother age	Below 20 years	63 (2.4%)	105 (2.9%)	168 (2.7%)
	Between 20 and 44 years	1939 (74.3%)	2743 (76.4%)	4682 (75.5%)
	35 years or more	607 (23.3%)	741 (20.6%)	1348 (21.7%)
Maternal education	No education	1363 (52.2%)	1779 (49.6%)	3142 (50.7%)
	Primary	432 (16.6%)	503 (14%)	935 (15.1%)
	Secondary	523 (20.0%)	760 (21.2%)	1283 (20.7%)
	Higher	291 (11.2%)	547 (15.2%)	838 (13.5%)
Maternal work	Yes	551 (21.2%)	416 (11.6%)	967 (15.6%)
	No	2051 (78.8%)	3171 (88.4%)	5229 (84.4%)
Birth in past year	No birth	1467 (56.2%)	2016 (56.2%)	3483 (56.2%)
	Yes	1142 (43.8%)	1573 (43.8%)	2715 (43.8%)
Last birth caesarean section	Yes	363 (13.9%)	724 (20.2%)	1087 (17.5%)
	No	2242 (86.1%)	2860 (79.7%)	5111 (82.5%)

*Household factor*				
Wealth index	Poorest	535 (20.5%)	737 (20.5%)	1272 (20.5%)
	Poorer	515 (19.7%)	886 (24.7%)	1401 (22.6%)
	Middle	474 (18.2%)	696 (19.4%)	1170 (18.9%)
	Richer	553 (21.2%)	632 (17.6%)	1185 (19.1%)
	Richest	532 (20.4%)	638 (17.8%)	1170 (18.9%)
Family size	Small-sized family	222 (8.5%)	323 (9%)	545 (8.8%)
	Medium-sized family	1416 (54.3%)	1856 (51.7%)	3272 (52.8%)
	Large-sized family	971 (37.2%)	1410 (39.3%)	2381 (38.4%)
Place of delivery	Home	1182 (45.3%)	1197 (33.4%)	2379 (38.4%)
	Health center	1427 (54.7%)	2392 (66.6%)	3819 (61.6%)
Region	Urban	1141 (43.7%)	1652 (46%)	2793 (45.1%)
	Rural	1468 (56.3%)	1937 (54%)	3405 (54.9%)
Province	Punjab	896 (34.3%)	764 (21.3%)	1660 (26.8%)
	Sindh	604 (23.2%)	691 (19.3%)	1295 (20.9%)
	KPK	442 (16.9%)	602 (16.8%)	1044 (16.8%)
	Baluchistan	231 (8.9%)	400 (11.1%)	631 (10.2%)
	GB	257 (9.9%)	233 (6.5%)	490 (7.9%)
	ICT	179 (6.9%)	220 (6.1%)	399 (6.4%)
	AJK	—	398 (11.1%)	398 (6.4%)
	FATA	—	281 (7.8%)	281 (4.5%)
Survey year	2012–2013	2609 (42.1%)	—	2609 (42.1%)
	2017–2018	—	3589 (57.9%)	3589 (57.9%)

**Table 2:** Determinants of double burden of malnutrition at household level of Pakistan.

**Variables**	**Categories**	**2012–2013** ^**a**^		**2017–2018** ^**b**^		**Total** ^**c**^	
		**Unadjusted odds (95% CI)**	**Adjusted odds (95% CI)**	**Unadjusted odds (95% CI)**	**Adjusted odds (95% CI)**	**Unadjusted odds (95% CI)**	**Adjusted odds (95% CI)**
*Child factors*							
Child age		1.01 (1.01–1.02)⁣^∗^	1.01 (1.01–1.02)⁣^∗^	1.02 (1.01–1.03)⁣^∗^		1.02 (1.01–1.02)⁣^∗^	1.01 (1.01–1.02)⁣^∗^
Child sex	Male	Ref		Ref		Ref	Ref
	Female	0.86 (0.72–1.02)		0.91 (0.78–1.05)		0.88 (0.79–0.99)⁣^∗^	0.86 (0.77–0.97)⁣^∗^
Birth order	Index child	Ref		Ref		Ref	
	Subsequent child	1.21 (0.97–1.49)		1.25 (1.03–1.51)⁣^∗^		1.24 (1.07–1.42)⁣^∗^	
Birth size	Average	Ref	Ref	Ref	Ref	Ref	Ref
	Small	0.65 (0.46–0.92)⁣^∗^	0.71 (0.49–1.04)	1.24 (1.02–1.52)⁣^∗^	1.35 (1.11–1.69)⁣^∗^	1.22 (1.05–1.41)⁣^∗^	1.29 (1.11–1.51)⁣^∗^
	Large	1.17 (0.94–1.46)	1.22 (0.96–1.54)	0.81 (0.59–1.11)	0.78 (0.56–1.07)	0.74 (0.58–0.93) ⁣^∗^	0.76 (0.59–0.96)⁣^∗^

*Maternal factors*							
Maternal age (categorical)	Below 20 years	Ref		Ref		Ref	
	Between 20 and 34 years	1.59 (0.84–3.00)		0.95 (0.61–1.50)		0.86 (0.59–1.24)	
	35 years or more	2.02 (1.05–3.88)⁣^∗^		1.00 (0.67–1.61)		1.15 (1.01–1.32)⁣^∗^	
Maternal education	No education	Ref	Ref	Ref	Ref	Ref	Ref
	Primary	0.96 (0.76–1.21)	1.31 (1.01–1.71)⁣^∗^	0.66 (0.52–0.84)⁣^∗^	0.75 (0.59–0.96)⁣^∗^	0.81 (0.68–0.94)⁣^∗^	1.02 (0.85–1.22)
	Secondary	0.49 (0.38–0.63)⁣^∗^	0.75 (0.56–1.01)	0.65 (0.53–0.81)⁣^∗^	0.79 (0.64–0.99)⁣^∗^	0.58 (0.50–0.68)⁣^∗^	0.84 (0.70–1.02)
	Higher	0.41 (0.29–0.57)⁣^∗^	0.69 (0.47–1.03)	0.37 (0.28–0.49)⁣^∗^	0.45 (0.34–0.60)⁣^∗^	0.38 (0.31–0.47)⁣^∗^	0.60 (0.47–0.77)⁣^∗^
Working status	No	Ref		Ref		Ref	
	Yes	1.32 (1.08–1.62)⁣^∗^		0.93 (0.73–1.18)		1.18 (1.01–1.37)⁣^∗^	
Birth in last year	No	Ref	Ref	Ref	Ref	Ref	Ref
	Yes	0.54 (0.45–0.65)⁣^∗^	0.58 (0.47–0.71)⁣^∗^	0.57 (0.49–0.67)⁣^∗^	0.71 (0.60–0.85)⁣^∗^	0.56 (0.50–0.63)⁣^∗^	0.67 (0.59–0.77)⁣^∗^
Last birth C-section	No	Ref		Ref		Ref	
	Yes	0.71 (0.54–0.92)⁣^∗^		0.69 (0.56–0.85)⁣^∗^		0.68 (0.58–0.81)⁣^∗^	

*Household factors*							
Wealth index	Poorest	Ref	Ref	Ref		Ref	Ref
	Poorer	0.64 (0.49–0.83)⁣^∗^	0.91 (0.68–1.21)	0.87 (0.71–1.08)		0.76 (0.64–0.91)⁣^∗^	0.87 (0.73–1.04)
	Middle	0.73 (0.56–0.95)⁣^∗^	1.04 (0.77–1.41)	0.71 (0.56–0.91)⁣^∗^		0.72 (0.61–0.85)⁣^∗^	0.94 (0.77–1.14)
	Richer	0.62 (0.48–0.81)⁣^∗^	0.92 (0.67–1.26)	0.60 (0.47–0.77)⁣^∗^		0.62 (0.52–0.74)⁣^∗^	0.82 (0.66–1.01)
	Richest	0.36 (0.27–0.47)⁣^∗^	0.61 (0.42–0.88)⁣^∗^	0.45 (0.35–0.59)⁣^∗^		0.41 (0.34–0.51)⁣^∗^	0.65 (0.51–0.82)⁣^∗^
Place of delivery	Home	Ref		Ref		Ref	
	Hospital/clinic	0.66 (0.56–0.78)⁣^∗^		0.79 (0.68–0.93)⁣^∗^		0.71 (0.64–0.81)⁣^∗^	
Family size	Small-sized family	Ref		Ref		Ref	
	Medium-sized family	1.24 (0.89–1.73)		1.10 (0.82–1.46)		1.05 (0.85–1.31)	
	Large-sized family	0.95 (0.72–1.37)		1.31 (0.98–1.76)		1.28 (1.03–1.59)⁣^∗^	

*Community factors*							
Region	Rural	Ref		Ref		Ref	
	Urban	0.91 (0.76–1.08)⁣^∗^		0.82 (0.71–0.96)⁣^∗^		0.85 (0.76–0.96)⁣^∗^	
Province	Islamabad	Ref	Ref	Ref	Ref	Ref	Ref
	Baluchistan	9.39 (5.73–15.37)⁣^∗^	7.62 (4.49–12.92)⁣^∗^	2.02 (1.34–3.05)⁣^∗^	1.59 (1.03–2.43)⁣^∗^	3.69 (2.71–5.03)⁣^∗^	2.83 (2.04–3.94)⁣^∗^
	Gilgit Baltistan	1.42 (0.84–2.38)	1.05 (0.60–1.82)	1.17 (0.73–1.89)	1.08 (0.66–1.76)	1.27 (0.90–1.81)	0.96 (0.66–1.38)
	Khyber Pakhtunkhwa	2.09 (1.31–3.39)⁣^∗^	1.64 (1.01–2.68)⁣^∗^	1.64 (1.11–2.44)⁣^∗^	1.43 (0.95–2.14)	1.82 (1.35–2.46)⁣^∗^	1.51 (1.11–2.06)⁣^∗^
	Punjab	1.79 (1.14–2.79)⁣^∗^	1.46 (0.92–2.34)	1.23 (0.83–1.83)	1.13 (0.76–1.69)	1.48 (1.11–1.98)⁣^∗^	1.21 (0.89–1.64)
	Sindh	2.76 (1.76–4.33)⁣^∗^	2.29 (1.42–3.69)⁣^∗^	1.61 (1.09–2.38)⁣^∗^	1.44 (0.96–2.15)	2.07 (1.54–2.77)⁣^∗^	1.69 (1.24–2.31)⁣^∗^
	AJK	—	—	1.09 (0.71–1.68)	1.05 (0.68–1.63)	1.19 (0.82–1.17)	1.17 (0.79–1.73)
	FATA	—	—	2.91 (1.89–4.43)⁣^∗^	2.34 (1.51–3.65)⁣^∗^	3.17 (2.21–4.54)⁣^∗^	2.72 (1.85–4.01)⁣^∗^

*Periodic factors*							
Survey period	2012–2013					Ref	Ref
	2017–2018					0.81 (0.72–0.91)⁣^∗^	0.75 (0.66–0.85)⁣^∗^

^a^Odds of DBM at household level were adjusted with child age, birth order, birth in last year, wealth index, place of delivery, number of children below 5 years old, family size, region, and province.

^b^Odds of DBM at household level were adjusted with child age, mother education, birth in last year, number of children below 5 years old, and province.

^c^Odds of DBM at household level were adjusted with child age, birth order, birth in last year, wealth index, place of delivery, number of children below 5 years old, family size, region, and province.

⁣^∗^Significant association between the two variables with *p* value ≤ 0.05.

## Data Availability

The data that support the findings of this study are openly available in the DHS Program at https://dhsprogram.com/.

## Supporting Information

Supporting Information Additional supporting information can be found online in the Supporting Information section. The supporting file has a table, which presents the unadjusted odds of double burden of malnutrition among mother–child dyads of Pakistan for the years 2012–2013, 2017–2018, and of both.
