# Supplementary material for: Prevalence, Trends, and Determinants of Double Burden of Malnutrition Among the Mother–Child Dyads of Pakistan
Source: Int J Pediatr. 2025 Jul 9;2025:8816802. doi: 10.1155/ijpe/8816802 (PMC12267966; doi:10.1155/ijpe/8816802)
Supplement: Supporting Information — Additional supporting information can be found online in the Supporting Information section. The supporting file has a table, which presents the unadjusted odds of double burden of malnutrition among mother–child dyads of Pakistan for the years 2012–2013, 2017–2018, and of both. [file 8816802.f1.docx]

**Prevalence, Trends, and Determinants of Double Burden of Malnutrition among the mother-child dyads of Pakistan**

**Supplementary file-1:**

**S-1: Unadjusted odds of Double Burden of Malnutrition of mother-child dyads of Pakistan**

| Variables | Categories | 2012-2013 | 2017-2018 | Total |
| --- | --- | --- | --- | --- |
| **Child factors** | | | | |
| Child age |  | 1.01 (1.00 to 1.02) * | 1.01 (1.01 to 1.02) * | 1.01 (1.00 to 1.02) * |
| Child sex | Female  Male | Ref  1.12 (0.92 to 1.37) | Ref  1.12 (0.95 to 1.33) | Ref  1.12 (0.99 to 1.28) |
| Birth order |  | 1.05 (1.01 to 1.10) * | 1.04 (1.00 to 1.07) * |  |
| Birth size | Average  Small  Large | Ref  1.10 (0.85 to 1.42)  0.77 (0.52 to 1.16) | Ref  1.07 (0.85 to 1.33)  1.05 (0.76 to 1.44) | Ref  1.08 (0.91 to 1.28)  0.93 (0.72 to 1.19) |
| **Maternal factors** | | | | |
| Maternal age (numerical) |  | 1.02 (1.00 to 1.03) * | 1.01 (1.00 to 1.02) * | 1.01 (1.00 to 1.02) * |
| Maternal age  (categorical) | 15-19 years  20-24 years  25-29 years  30-34 years  35-39 years  40-44 years  45-49 years | Ref  1.36 (0.62 to 2.95)  1.26 (0.58 to 2.73)  1.54 (0.71 to 3.31)  2.03 (0.93 to 4.41)  1.23 (0.51 to 2.93)  1.71 (0.62 to 4.74) | Ref  1.98 (1.03 to 3.79) *  1.96 (1.03 to 3.72) *  1.99 (1.04 to 3.80) *  2.07 (1.06 to 4.01) *  2.55 (1.24 to 5.26) *  3.06 (1.28 to 7.34) * | Ref  1.70 (1.03 to 2.79) *  1.65 (1.01 to 2.73) *  1.79 (1.09 to 2.94) *  2.08 (1.25 to 3.43) *  1.88 (1.08 to 3.27) *  2.38 (1.23 to 4.51) * |
| Maternal education | No education  Primary  Secondary  Higher | Ref  1.08 (0.82 to 1.42)  0.84 (0.64 to 1.18)  0.79 (0.56 to 1.13) | Ref  0.68 (0.52 to 0.90) *  0.92 (0.74 to 1.14)  0.62 (0.47 to 0.81) * | Ref  0.84 (0.70 to 1.02)  0.89 (0.75 to 1.06)  0.68 (0.55 to 0.85) * |
| No. of children delivered |  | 1.05 (1.01 to 1.95) * | 1.04 (1.00 to 1.08) * | 1.04 (1.01 to 1.07) * |
| Birth in last year | No  Yes | Ref  0.66 (0.53 to 0.81) * | Ref  0.56 (0.47 to 0.67) * | Ref  0.60 (0.52 to 0.68) * |
| Last birth C-section | No  Yes | Ref  1.23 (0.93 to 1.62) | Ref  0.80 (0.65 to 1.01) | Ref  0.94 (0.80 to 1.12) |
| Marital status | Other**  Married | Ref  0.54 (0.22 to 1.31) | Ref  0.78 (0.39 to 1.54) | Ref  0.68 (0.40 to 1.17) |
| **Household factors** | | | | |
| Wealth index | Poorest  Poorer  Middle  Richer  Richest | Ref  0.77 (0.55 to 1.07)  1.08 (0.79 to 1.49)  1.17 (0.86 to 1.58)  0.82 (0.59 to 1.13) | Ref  0.91 (0.71 to 1.15)  0.90 (0.71 to 1.15)  0.88 (0.68 to 1.15)  0.71 (0.54 to 0.93) * | Ref  0.86 (0.71 to 1.05)  0.97 (0.79 to 1.19)  1.00 (0.82 to 1.22)  0.75 (0.61 to 0.93) * |
| Place of delivery | Home  Hospital/clinic | Ref  1.10 (0.90 to1.35) | Ref  0.91 (0.71 to 1.08) | Ref  0.99 (0.87 to 1.13) |
| No. of children below five years |  | 0.88 (0.81 to 0.95) * | 0.92 (0.87 to 0.98) * | 0.91 (0.87 to 0.95) * |
| Family size |  | 1.00 (0.98 to 1.02) | 0.99 (0.98 to 1.01) | 1.00 (0.98 to 1.01) |
| **Community factors** | | | | |
| Region | Rural  Urban | Ref  1.26 (1.03 to 1.54) * | Ref  1.03 (0.87 to 1.21) | Ref  1.12 (0.98 to 1.27) |
| Province | Islamabad  Baluchistan  Gilgit Baltistan  Khyber Pakhtunkhwa  Punjab  Sindh  AJK  FATA | Ref  3.04 (1.79 to 5.15) *  0.94 (0.52 to 1.69)  1.77 (1.07 to 2.93) *  1.45 (0.91 to 2.35) *  1.39 (0.85 to 2.29)  -  - | Ref  1.31 (0.85 to 2.01)  0.68 (0.40 to 1.14)  1.33 (0.89 to 1.98)  1.09 (0.73 to 1.62)  1.02 (0.68 to 1.53)  0.70 (0.44 to 1.11)  1.94 (1.25 to 2.99) * | Ref  1.84 (1.32 to 2.55) *  0.77 (0.52 to 1.13)  1.50 (1.09 to 2.50) *  1.21 (0.89 to 1.64)  1.16 (0.85 to 1.58)  0.83 (0.55 to 1.24)  2.28 (1.57 to 3.33) * |
